# Molecular and metabolic drivers of oil quality and bioenergy potential in *Jatropha curcas*

**DOI:** 10.1007/s00425-026-05159-9

**Published:** 2026-09-25

**Authors:** Daniela de Argollo Marques, Kleber Alves Gomes, Roseli Aparecida Ferrari, Cássia Regina Limonta Carvalho, Walter José Siqueira, Nicolas Carels, Marie-Anne Van Sluys

**Affiliations:** 1 Centro de Recursos Genéticos Vegetais, Instituto Agronômico (IAC), Campinas, São Paulo, 13075-630 Brazil; 2 Centro de Ciência e Qualidade de Alimentos (CCQA), Instituto de Tecnologia de Alimentos (ITAL), Campinas, São Paulo, Brazil; 3 Centro de Desenvolvimento Tecnológico em Saúde (CDTS), Fundação Oswaldo Cruz (Fiocruz), Rio de Janeiro, Brazil; 4 Instituto de Biociências, Laboratório de Genômica e Elementos Transponíveis (GaTE), Universidade de São Paulo, São Paulo, Brazil

**Keywords:** Fatty acid composition, Higher heating value, Oleic acid, Precision breeding, Seed development, Specialized diterpenoid metabolism

## Abstract

**Main conclusion:**

Combining biochemical, molecular, and multivariate analyses identified candidate signatures and L10P41 as a promising bioenergy ideotype, supporting targeted selection for oil quality and bioenergy performance in *Jatropha curcas*.

**Abstract:**

*Jatropha curcas* L. is a promising bioenergy crop, but its genetic improvement requires a deeper understanding of the molecular and metabolic factors underlying variation in oil quality, fatty acid (FA) composition, and phorbol ester (PE) accumulation. We characterized FA composition during seed development and evaluated mature-seed oil content, PE concentration, higher heating value (HHV), and the expression of *KASIII*, *MFP2*, and *JcBAHD-AT* in 12 contrasting elite breeding genotypes. Stage-specific principal component analysis (PCA), complemented by hierarchical cluster analysis (HCA), revealed developmentally dynamic variation in FA composition and genotype relationships. Comparison with biochemical and expression data indicated associations among lipid composition, candidate-gene expression, PE concentration, and energy performance. The L10P41 genotype combined an oleic acid-enriched profile, relatively high oil content, elevated HHV, and distinct *KASIII* and *MFP2* expression patterns, emerging as a promising bioenergy ideotype. Variation in HHV among genotypes, including relatively high values in some genotypes with comparatively low oil content, indicated that energy performance cannot be inferred from total oil content alone and may also be associated with differences in oil composition. The putative BAHD acyltransferase gene *JcBAHD-AT* exhibited genotype- and stage-specific expression patterns consistent with a potential role in specialized diterpenoid metabolism; however, its relationship with PE accumulation remains associative and requires functional validation. Genotypes combining high oil content with comparatively low PE concentrations further indicated that these traits can coexist and are not necessarily tightly coupled. Overall, integrating molecular, biochemical, and multivariate analyses revealed candidate signatures associated with oil quality and bioenergy potential, advancing the identification of promising genotypes for targeted selection and precision breeding in *J. curcas*.

**Supplementary Information:**

The online version contains supplementary material available at https://doi.org/10.1007/s00425-026-05159-9.

## Introduction

Amid increasing concerns about environmental sustainability, climate change, energy security, and competition between food and fuel production, renewable biofuels derived from non-edible feedstocks, such as *Jatropha curcas* L. (Euphorbiaceae), have emerged as promising alternatives to fossil fuels (Riayatsyah et al. 2022; Ruatpuia et al. 2024). Among the perennial oilseed crops, *J. curcas* has attracted considerable attention because of its non-edible oil, favorable fatty acid (FA) composition, adaptability to tropical and subtropical environments, and potential use as a biodiesel feedstock (Riayatsyah et al. 2022; Ruatpuia et al. 2024). These characteristics support its potential contribution to renewable energy production and the transition toward sustainable bio-based economies. Moreover, the increasing demand for plant-derived oils emphasizes the need to develop oilseed crops with enhanced productivity, improved oil quality, and greater metabolic efficiency (Sagun et al. 2023; Gao et al. 2025).

*J. curcas*, commonly known as physic nut, is a perennial non-edible oilseed crop characterized by seed oil contents commonly ranging from 30 to 40% and a favorable FA profile, frequently containing a high proportion of oleic acid (~ 45%) (Carels 2009; Islam et al. 2022; Ruatpuia et al. 2024**).** However, the commercial development of the crop remains constrained by low and variable seed productivity, particularly in unimproved germplasm and under suboptimal growing conditions (Achten et al. 2010; Riayatsyah et al. 2022; Ruatpuia et al. 2024). Recent breeding studies have identified promising families, parents, and clones based on productive traits, demonstrating the potential of targeted selection to support the genetic improvement of *J. curcas* (Silva et al. 2025). FA composition is a major determinant of biodiesel quality because it influences viscosity, cetane number, oxidative stability, cold-flow properties**,** and combustion performance (Knothe 2008; Ramos et al. 2009; de Argollo Marques et al. 2013; Nithyananda et al. 2024). Furthermore, the physicochemical and energetic properties of *J. curcas* seed oil vary according to genotype and plant age, with reported higher heating values (HHVs) ranging from approximately 39.8 to 41.8 MJ kg⁻^1^ (Islam et al. 2022).

Despite these advantages, *J. curcas* remains a partially domesticated species and continues to face important agronomic constraints, including a low female-to-male flower ratio, susceptibility to pests and diseases (de Argollo Marques et al. 2013), and seed toxicity associated with the accumulation of phorbol esters (PEs) (Kumar et al. 2012). Nevertheless, naturally occurring non-toxic genotypes (< 0.05 mg g⁻^1^ PE), particularly from Mexico, represent valuable genetic resources because they combine bioenergy potential with the possibility of using the protein-rich seed cake for animal feed (Makkar et al. 1997; Sanghamitra et al. 2014). In contrast, most *J. curcas* accessions accumulate PE concentrations above this threshold. The extensive variation in productivity, oil content, FA composition, and PE concentration therefore constitutes an important basis for breeding programs aimed at improving seed oil quality and bioenergy potential (de Argollo Marques et al. 2013).

Conventional breeding has contributed to improvements in yield and oil composition; however, progress remains constrained by biological limitations, including long generation cycles, incomplete domestication, and variable responses to biotic and abiotic stresses (King et al. 2009). Recent studies have characterized transcriptional, physiological, and molecular responses of *J. curcas* to abiotic stresses, particularly salinity, and have identified candidate gene families potentially involved in broader stress adaptation (de Lima Cabral et al. 2020; Pompelli et al. 2022; da Silva et al. 2024). More broadly, metabolomics-based approaches have identified stress-responsive metabolites and gene–metabolite relationships as promising resources for understanding drought adaptation and supporting the development of stress-resilient crops (Raza et al. 2025)**.** Regarding biotic stress, heterologous expression of *JcWRKY2* enhanced resistance to *Macrophomina phaseolina* in transgenic tobacco, supporting a potential role for this transcription factor in pathogen defense (Dabi et al. 2020).

Integrating genomic resources with biochemical phenotyping provides new opportunities to accelerate genetic improvement by identifying genes involved in lipid biosynthesis, FA remodeling, and specialized diterpenoid metabolism associated with PE production and plant defense (Sato et al. 2011). Transcriptomic analyses of developing seeds have identified numerous candidate genes involved in lipid accumulation and PE-related pathways (Costa et al. 2010; Gu et al. 2012). Within a systems biology framework, integrating gene expression profiles with metabolic phenotyping can reveal coordinated molecular networks underlying complex bioenergy traits (Han et al. 2024; Raza et al. 2025). Rather than evaluating individual metabolic pathways in isolation, this approach can uncover regulatory relationships among carbon partitioning, FA biosynthesis, lipid remodeling, and specialized metabolism during seed development.

Given the substantial natural variation in FA composition among *J. curcas* genotypes (King et al. 2013), multivariate approaches such as principal component analysis (PCA) and hierarchical cluster analysis (HCA) with heatmap visualization are valuable for characterizing complex variation in FA profiles and genotype relationships. PCA can summarize the major dimensions of variation in FA composition, whereas HCA can complement this analysis by grouping genotypes according to similarities in the same multivariate FA dataset (Eisen et al. 1998; Pang et al. 2021). Applied across developmental stages, these complementary approaches can help determine whether the multivariate structure of FA composition and relationships among genotypes remain stable or change during seed development, providing additional information for the characterization of oil-quality diversity and germplasm selection.

Recent advances in plant systems biology indicate that carbon allocation, cellular energy signaling, transcriptional regulation, and environmental cues interact to shape primary and specialized metabolism (Han et al. 2024; Raza et al. 2025). The resulting metabolic phenotypes emerge from interconnected networks rather than from individual genes acting independently (Baud and Lepiniec 2010; Bates et al. 2013; Li-Beisson et al. 2013; Vranová et al. 2013). Integrating molecular and metabolic information can therefore clarify associations among regulatory processes, seed composition, and bioenergy-related traits. This systems-level perspective is particularly relevant for *J. curcas*, in which natural variation in oil content and FA composition is associated with fuel properties, bioenergy potential, and breeding value (Alburquerque et al. 2017; Kumar and Das 2018).

Despite significant advances in the characterization of oil content, FA composition, PE accumulation, and genes associated with lipid biosynthesis and specialized metabolism (Costa et al. 2010; Gu et al. 2012; King et al. 2013), comprehensive studies jointly evaluating gene expression, biochemical composition, and bioenergy-related traits across contrasting *J. curcas* genotypes remain limited. Consequently, the associations between gene expression patterns and lipid composition, oil quality, energy potential, and PE accumulation remain incompletely resolved, constraining the identification of molecular and metabolic signatures that could support genotype selection and targeted breeding.

In the present study, we integrated FA profiling, PE quantification, HHV determination, and expression analysis of *KASIII*, *MFP2*, and *JcBAHD-AT* to investigate molecular, metabolic and energetic features associated with oil quality and bioenergy potential during seed development in contrasting elite genotypes of *J. curcas*. The stage-specific PCA and complementary HCA were applied to FA composition to characterize multivariate variation in lipid profiles and genotype relationships throughout seed development. This integrative framework enabled the simultaneous evaluation of gene expression, biochemical traits, and energy performance, providing a basis for examining their relationships and identifying candidate signatures associated with seed quality and bioenergy potential.

Accordingly, three candidate genes were selected because they represent complementary processes involved in FA biosynthesis, FA turnover, and putative specialized metabolism during seed development. *KASIII*, a single-copy gene in *J. curcas* (Hirakawa et al. 2012), catalyzes the initial condensation of acetyl-CoA and malonyl-ACP in plastidial de novo FA biosynthesis (Li-Beisson et al. 2013). *MFP2* encodes a multifunctional protein involved in peroxisomal β-oxidation and FA turnover (Arent et al. 2010). *JcBAHD-AT* encodes a putative BAHD acyltransferase and shares approximately 90% amino acid sequence similarity with a predicted 3′-*N*-debenzoyl-2′-deoxytaxol N-benzoyltransferase from *Ricinus communis*. This gene was included to investigate potential associations between its expression, specialized diterpenoid metabolism, and PE accumulation, in the context of casbene-related diterpenoid biosynthesis described in *J. curcas* (Nakano et al. 2012); however, its biochemical function and involvement in PE biosynthesis remain unvalidated.

## Materials and methods

### Plant material, genotype selection and growth conditions

Twelve elite *J. curcas* genotypes were selected from the breeding program of Instituto Agronômico (IAC), São Paulo, Brazil. These elite breeding materials are maintained in the IAC germplasm collection. Although they are not currently available through a public germplasm database, access may be considered through collaborative research agreements with the IAC. They were selected based on contrasting oil content, PE concentration, and agronomic traits to enable comparative metabolic and molecular analyses. Detailed information on genotype origin and selection criteria is provided in Table 1.

**Table 1:** Characterization of the twelve elite *Jatropha curcas* genotypes evaluated in this study, including their origin, oil content, phorbol ester (PE) concentration, and additional agronomic traits used as selection criteria in the IAC breeding program. The selected genotypes represent broad phenotypic diversity for integrated molecular and metabolic analyses

Genotype ID	Origin/Pedigree	Oil content (%)	Phorbol ester (mg g⁻^1^)	Other selection traits
L13P56	Adamantina, São Paulo, Brazil	21.0	1.6	Reduced plant stature
L4P17	Adamantina, APTA, São Paulo, Brazil	37.2	1.6	High fruit yield, high oil content; fungal resistance
L12P52	Minas Gerais, Brazil	35.0	0.7	High fruit yield, high oil content; low phorbol ester concentration
L3P29	Adamantina, São Paulo, Brazil	25.0	0.8	Reduced plant stature; low phorbol ester concentration
L10P41	Ataliba Leonel, São Paulo, Brazil	34.9	1.7	High oil content; high fruit yield; moderate plant stature; fungal resistance
L3P24	Adamantina, São Paulo, Brazil	18.5	0.9	Reduced plant stature; high fruit yield
L4P36	Adamantina, APTA, São Paulo, Brazil	31.8	2.0	Moderate fruit yield; moderate plant stature; fungal resistance
L4P49	Fortaleza, Ceará, Brazil	32.0	0.5	High fruit yield; moderate oil content; low phorbol ester concentration
L3P50	Iraçê, Bahia, Brazil	34.8	0.6	High oil content; low phorbol ester concentration; high fruit yield
L9P32	Janaúba, Minas Gerais, Brazil	33.0	1.2	High fruit yield
L4P23	Adamantina, APTA, São Paulo, Brazil	32.0	1.3	High fruit yield
L7P30	Mato Grosso do Sul, Brazil	33.6	~1.2	High fruit yield, high oil content

Plants were grown under field conditions in the experimental orchard at Fazenda Santa Elisa (IAC, Campinas, São Paulo, Brazil) under uniform agronomic management and the same edaphoclimatic conditions for all genotypes. All plants received the same fertilization regimen, including basal and top-dressing fertilization according to the IAC breeding program recommendations. Irrigation was applied only during orchard establishment, after which the plants relied exclusively on natural rainfall. All genotypes were cultivated in the same experimental area to minimize environmental variation among treatments. The meteorological conditions recorded at the experimental site on the developing-seed sampling dates, including daily rainfall and relative humidity, are summarized in Table 2.

**Table 2:** Environmental conditions recorded during the sampling period at Fazenda Santa Elisa, Instituto Agronômico (IAC), Campinas, São Paulo, Brazil. Meteorological data were recorded by the weather station located at Fazenda Santa Elisa, IAC, on the respective sampling date of each developmental stage

Developmental stage	Fruit length (mm)	Rainfall (mm)	Relative humidity (07:00 h, %)	Relative humidity (13:00 h, %)
Stage 1	10–15	12	100	28
Stage 2	20–25	12	100	25
Stage 3	30–40	10	81	64

**Fig. 1:**
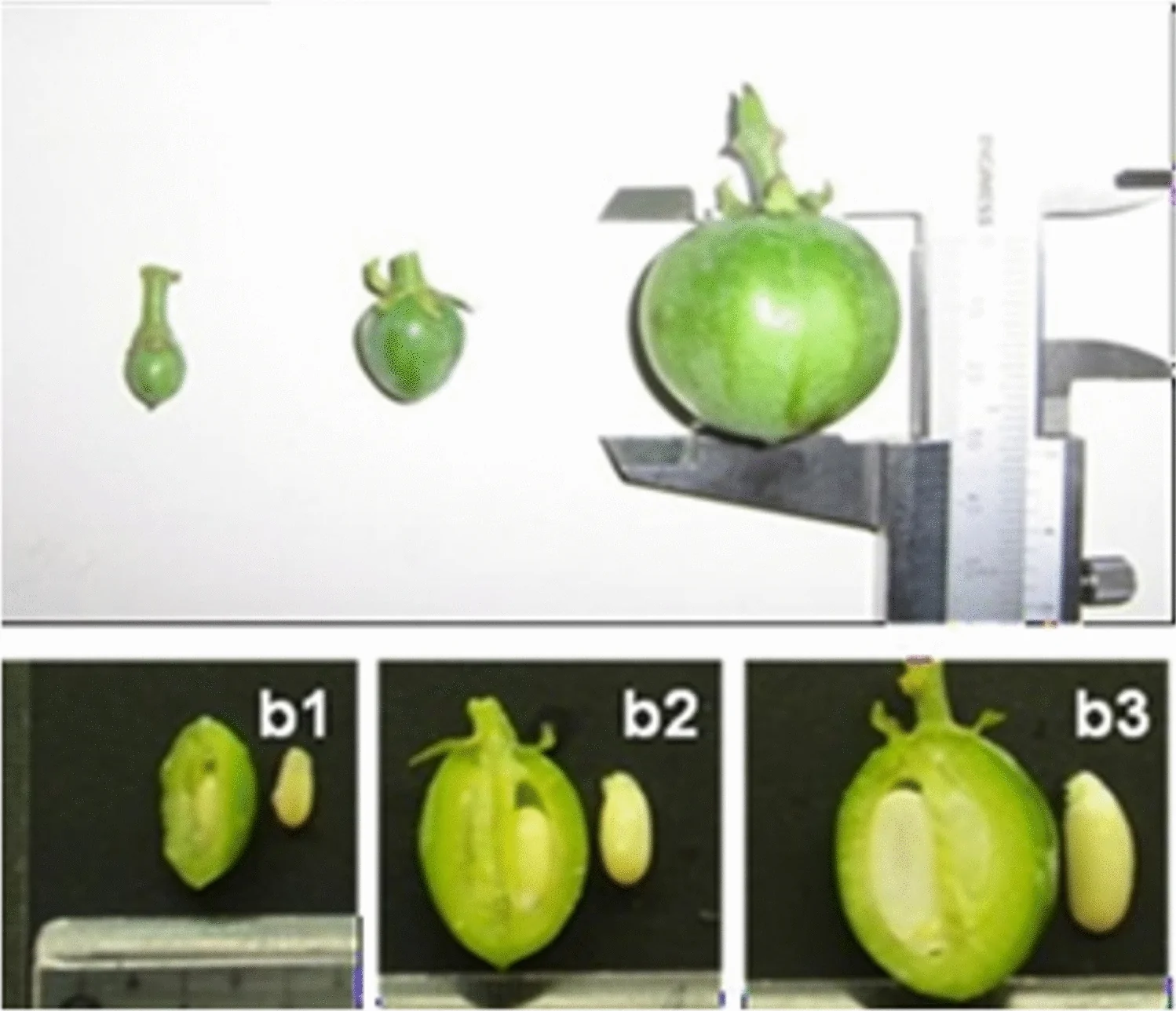
Morphological characterization of fruit developmental stages in *Jatropha curcas*. Three fruit developmental stages were defined according to fruit length (longitudinal axis): 10–15 mm, 20–25 mm, and 30–40 mm, corresponding to 15, 19, and 23 days after pollination (DAP), respectively. Longitudinal sections show progressive internal tissue development and seed formation. Scale bars are indicated where applicable

Sampling followed the phenological development of the species in southeastern Brazil (de Argollo Marques et al. 2013), from flowering initiation (October) to fruit maturation (December). Three fruit developmental stages were defined based on longitudinal fruit length (10–15 mm, 20–25 mm, and 30–40 mm), corresponding to approximately 15, 19, and 23 days after pollination (DAP), respectively. These stages represent successive phases of seed development. The morphological characteristics of the fruit and seeds at the three developmental stages are shown in Fig. 1. For each genotype, three independent biological samples were collected at each tissue or developmental-stage sampling event, comprising three different flower samples and three distinct seed samples at each of Stages 1, 2, and 3. Flowers and developing seeds at the three stages were collected during their respective flowering and seed-development periods. At each sampling event, all genotypes were collected on the same date and processed using the same procedures to minimize short-term environmental and handling variation. Meteorological conditions at the experimental site on the developing-seed sampling dates are presented in Table 2. For oil content, PE concentration, and HHV determination, fully mature fruits exhibiting yellow-to-brown coloration were collected at 41–45 DAP, and their seeds were used for subsequent analyses. For each genotype, these analyses were performed using three independently collected mature-seed samples (*n* = 3). Three independent fully expanded leaf samples (~ 8 cm in length) were collected and used as the calibrator tissue for gene expression analyses. All organs were measured along the longitudinal axis (apex to base) using a digital caliper to ensure consistency across samples. Immediately after harvesting, the samples intended for gene expression and FA composition analyses were flash-frozen in liquid nitrogen, transported under cryogenic conditions, homogenized to a fine powder, and stored at −80 °C until further analysis. The samples from all genotypes were processed following the same handling and storage procedures to preserve RNA integrity and metabolite composition.

### Gene identification, annotation and selection criteria

The three target genes analyzed in this study were *KASIII* (gene ID: Jcr4S00903.20), *MFP2* (gene ID: Jcr4S00616.80), and *JcBAHD-AT* (gene ID: Jcr4S00504.110), identified according to the annotated gene models available in the Jatropha genome database (Kazusa DNA Research Institute 2026). Functional annotation was further supported by sequence similarity information available in NCBI GenBank and UniProt databases, together with the presence of conserved sequence motifs characteristic of the respective protein families. *Ubiquitin* (gene ID: Jcr4S10519.50) was used as the endogenous reference gene for RT-qPCR normalization. Gene-specific primers were designed based on the coding regions of each gene, generating amplicons of 174 bp (*KASIII*), 61 bp (*MFP2*), 112 bp (*JcBAHD-AT*), and 150 bp (*ubiquitin*) (Table 3). *KASIII* encodes a β-ketoacyl-ACP synthase III involved in the initial condensation step of plastidial FA biosynthesis. *MFP2* encodes multifunctional protein 2, a peroxisomal enzyme involved in FA β-oxidation and lipid turnover. *JcBAHD-AT* encodes a putative BAHD acyltransferase, and its annotation was further supported by sequence similarity and conserved BAHD-related motifs. The selection of *KASIII*, *MFP2*, and *JcBAHD-AT* was based on their complementary predicted functions in lipid metabolism and specialized metabolite biosynthesis during seed development. *KASIII* was selected as a candidate associated with FA biosynthesis and carbon allocation toward storage lipid accumulation. *MFP2* was selected due to its role in FA degradation and lipid remodeling during seed maturation. Although broader KAS, MFP, and BAHD gene families contain multiple members, the objective of this study was not to characterize complete gene families, but rather to investigate biologically relevant candidate genes with predicted functions in metabolic pathways potentially influencing seed oil traits. *JcBAHD-AT* was selected as a candidate potentially associated with specialized diterpenoid metabolism. Its sequence analysis revealed 83% amino acid identity and approximately 90% similarity with a putative 3′-*N*-debenzoyl-2′-deoxytaxol N-benzoyltransferase from *Ricinus communis* (UniProt accession B9S966). Therefore, the functional annotation of *JcBAHD-AT* remains putative and requires further experimental validation.

**Table 3:** Primer sequences, gene identifiers from the Kazusa Jatropha genome database, coding sequence (CDS) length, and amplicon sizes used for RT-qPCR analysis. Ta, annealing temperature. *Ubiquitin* was used as the endogenous reference gene

	Gene ID (Kazusa)	CDS length (bp)	Primer sequence (5′–3′)	Amplicon (bp)	Ta (°C)
*KASIII*	Jcr4S00903.20	1203	F: CCAAGTTAGACCAGCTCCAA	174	60
			R: GCAGTTGCGACACGATTAGA		
*MFP2*	Jcr4S00616.80	2475	F: GGACAATCTCCAAAAGTGGCATA	61	60
			R: CGGATCATTGGAGCCCATT		
*JcBAHD-AT*	Jcr4S00504.110	1338	F: TGAGTGGCTTCACAGATTGG	112	60
			R: GGTACAACACTTCCAAGTAG		
*Ubiquitin*	Jcr4S10519.50	—	F: AAGATGTTGGAGGTGGTGTC	150	60
			R: GGTGTACCACTTCTGTATCC		

### Gene expression analysis

Genomic DNA contamination was removed using Turbo DNase (Ambion), and RNA integrity was verified by agarose gel electrophoresis. Total RNA was extracted from leaf tissue, flower buds, and developing seeds of *J. curcas*. RNA from developing seeds (1 g of tissue) was isolated using the PureLink RNA Mini Kit (Ambion), whereas RNA from leaves and flower buds was extracted using the lithium chloride method (Sambrook et al. 1989). RNA concentration and purity were assessed using a NanoVue Plus spectrophotometer (GE Healthcare). First-strand cDNA was synthesized from 1 µg of total RNA using SuperScript III Reverse Transcriptase (Thermo Fisher Scientific), according to the manufacturer’s instructions.

### qPCR primer design and validation

Gene-specific primers for *KASIII*, *MFP2*, and *JcBAHD-AT* were designed from their coding sequences obtained from the Kazusa genome annotation and validated by BLAST searches against NCBI to ensure target specificity. The primer design was performed using Primer Designer 2.0, targeting amplicons of 60–200 bp suitable for RT-qPCR analysis. Specificity of amplification was confirmed by melting curve analysis and agarose gel electrophoresis. Primer sequences, gene identifiers, coding sequence (CDS) lengths, amplicon sizes, and annealing temperatures are listed in Table 3.

### qPCR conditions and expression analysis

Quantitative PCR (qPCR) reactions were performed in a final volume of 20 µL containing 10 µL of SYBR Green PCR Master Mix, 4 µL of primer mixture (forward and reverse primers, 200 nM each) , 5 µL of cDNA containing 50 ng of total cDNA, and 1 µL of nuclease-free water. Amplifications were performed using a 7300 Real-Time PCR System (Applied Biosystems) under the following cycling conditions: 55 °C for 5 min, followed by 95 °C for 10 min and 40 cycles of 95 °C for 15 s and 60 °C for 1 min, followed by a dissociation step. Amplification specificity was verified by melting-curve analysis. All reactions were performed in technical triplicate. Relative transcript abundance was determined using the 2^⁻ΔΔCt^ method (Livak and Schmittgen 2001; Bookout and Mangelsdorf 2003), with *ubiquitin* used as the endogenous reference gene for normalization because of its stable expression across tissues and developmental stages. For each target gene, Ct values were normalized to *ubiquitin* and relative expression levels were calculated using leaf samples as the calibrator. Fold-change patterns were visualized using JColorGrid (Joachimiak et al. 2006).

### Primer efficiency assessment

Amplification efficiency was determined for all target-gene and reference-gene primer pairs using serial dilutions of pooled cDNA. Amplification efficiencies ranged from 83 to 100%, with correlation coefficients (*R*^2^) ≥0.98, supporting their suitability for relative expression analysis.

### Oil content and FA composition

To determine moisture and dry mass contents, the seed samples were initially weighed and dried in a forced-air oven at 60 °C for 16 h, followed by an additional drying step at 100 °C for 1 h. Seed oil content was determined by exhaustive extraction with 30 mL of *n*-hexane using a Butt extractor, according to Carvalho et al. (1990). After extraction, the solvent was removed under reduced pressure using a rotary evaporator (Büchi R-114). The extracted oil was subsequently dried in a ventilated oven at 105 °C for 6 h, cooled in a desiccator, and weighed on an analytical balance to determine the final oil mass. Oil content, initially expressed on a dry-weight basis, was converted to a fresh-weight basis using the corresponding moisture content. Fatty acid methyl esters (FAMEs) were prepared by successive esterification and transesterification according to Hartman and Lago (1973). FA composition was determined by gas chromatography with flame ionization detection (GC-FID) following AOAC Method 996.06 (AOAC International 2002), using a Shimadzu GC-2010 gas chromatograph equipped with an AOC-20i autosampler, a flame ionization detector (FID), and a CP-SIL 88 fused-silica capillary column (100 m × 0.25 mm i.d., 0.20 µm film thickness). The oven temperature program consisted of an initial temperature of 120 °C held for 2 min, followed by heating to 220 °C at 2.2 °C min⁻^1^ and then to 235 °C at 1.5 °C min⁻^1^, with a final holding time of 15 min. Hydrogen was used as the carrier gas at a flow rate of 1 mL min⁻^1^, whereas nitrogen was used as the make-up gas at 30 mL min⁻^1^. Injector and detector temperatures were maintained at 270 °C and 310 °C, respectively, and the injection volume was 1 µL. FAs were identified by comparing their retention times with authenticated FAME standards and quantified by chromatographic peak-area normalization. FA composition was expressed as the relative percentage of chromatographic peak area (% area) for each identified FA. These relative abundance data were subsequently used for PCA and HCA to investigate patterns of variation in FA composition among genotypes at each developmental stage.

### Principal component, hierarchical cluster, and heatmap analyses of FA composition

FA composition data, expressed as the relative abundance (%) of the identified FAs, were analyzed separately for each fruit developmental stage (Stages 1–3). The set of FA variables included in the multivariate analyses was defined separately for each developmental stage according to the FAs detected and their within-stage variability. Unidentified chromatographic peaks were excluded, as were variables with zero variance within a given stage because they could not be standardized. Accordingly, 13 FAs were retained at Stage 1, 15 at Stage 2, and 16 at Stage 3. The retained variables were C12:0, C14:0, C15:0, C16:0, C17:0, C18:0, C18:1ω9, C18:2ω6, C18:3ω3, C20:0, C22:0, C23:0, and C24:0 at Stage 1; these same FAs plus C20:1ω11 and C21:0 at Stage 2; and C12:0, C14:0, C15:0, C16:0, C16:1ω7, C17:0, C18:0, C18:1ω9, C18:2ω6, C18:3ω3, C20:0, C20:1ω11, C21:0, C22:0, C23:0, and C24:0 at Stage 3. At Stage 3, C10:0 and C11:0, detected only in genotype L3P24, were not included in the multivariate analysis to maintain the predefined analytical FA range of C12:0–C24:0. Before multivariate analyses, all variables were autoscaled (mean-centered and scaled to unit variance) to ensure equal weighting of individual FAs regardless of their relative absolute abundance. PCA was performed separately for each developmental stage in R (R Core Team 2026) using singular value decomposition (SVD) to summarize variation in FA composition among genotypes within each developmental stage. The first two principal components (PC1 and PC2), which accounted for the largest proportions of the total variance, were used to visualize genotype distribution and the contribution of individual FAs through their loading vectors. HCA was performed separately for each developmental stage using the same autoscaled matrices employed for PCA. Euclidean distance was used as the dissimilarity measure and hierarchical clustering was performed using Ward’s minimum variance method (Ward.D2). The number of clusters was selected by comparing the average silhouette width for solutions ranging from *k* = 2 to *k* = 5, with the solution yielding the highest average silhouette width retained for each developmental stage. This procedure resulted in two clusters for Stages 1 and 2 and three clusters for Stage 3. The HCA results were visualized as heatmaps with hierarchical dendrograms, with FA abundance patterns represented as row-wise standardized Z-scores.

### Higher heating value

Oil samples from contrasting *J. curcas* genotypes were extracted from seeds collected from fully mature yellow-to-brown fruits. For each genotype, the analyses were performed using three biological replicates (*n* = 3). Approximately 0.8–1.0 g of crude oil was weighed into a crucible and subjected to combustion in an oxygen bomb calorimeter to determine its energy content. The bomb was pressurized with oxygen to 30 atm by connecting the inlet valve to an O₂ cylinder. After pressurization, the cylinder was disconnected, and the bomb was immersed in the calorimetric vessel containing a known mass of water, which was continuously stirred to ensure uniform temperature distribution. A precision thermometer (± 0.01 °C) was used to record temperature readings at 1-min intervals for 5 min before ignition. After ignition, temperature measurements were recorded at 45, 60, 75, 90, 105, and 120 s, followed by additional readings at 1-min intervals until temperature stabilization or slight linear variation was observed. Temperature–time curves were generated and corrected following the comb-calorimetry procedure described by Demirbas (1998). HHV was calculated according to Eqs. (1) and (2) and expressed as kcal kg⁻^1^.

1 $$HHV=({C}^{*}.\Delta T-{e}^{*})/ m$$

2 $${e}^{*}={m}_{Fe}. PC(Fe)$$

where HHV is the higher heating value (kcal kg⁻^1^), C* is the thermal capacity of the calorimeter (cal °C⁻^1^), ΔT is the corrected temperature change (°C), e* is the heat released by combustion of the iron wire (cal), mFe is the mass of iron wire consumed (g), PC(Fe) is the calorific power of the iron wire (cal g⁻^1^), and m is the mass of the oil sample (g).

### Phorbol ester concentration

PE extraction and sample preparation were performed according to Makkar et al. (1997). PE concentration was determined by high-performance liquid chromatography (HPLC) using a Shimadzu Prominence system (Shimadzu, Kyoto, Japan) equipped with a diode-array detector and a LiChrospher 100 RP-18 reversed-phase column (125 × 4 mm, 5 µm particle size; Merck). The column temperature was maintained at 22 °C, the injection volume was 20 µL, and the mobile-phase flow rate was 1.3 mL min⁻^1^. Detection was performed at 280 nm. The mobile phase consisted of 0.175% phosphoric acid in deionized water (solvent A) and filtered and degassed acetonitrile (solvent B). The gradient program was as follows: 60% A and 40% B at 0 min, 50% A and 50% B at 10 min, 25% A and 75% B at 40 min, and 100% B at 55 min. The PE concentration was determined by external calibration using phorbol-12-myristate−13-acetate (TPA; Sigma-Aldrich) as the reference standard and expressed as TPA equivalents.

### Statistical analysis

Oil content and PE concentration in fully mature seeds were subjected to one-way analysis of variance (ANOVA), with genotype as the fixed factor. When significant differences were detected, means were grouped using the Scott–Knott test at *P* ≤ 0.05. For RT-qPCR analysis, each genotype–tissue combination was analyzed in technical triplicate. The mean Ct value of the three technical replicates was used to calculate ΔCt relative to the endogenous reference gene, *ubiquitin*, and ΔΔCt relative to the leaf calibrator. Relative gene expression was calculated using the 2⁻ΔΔCt method and expressed as fold change relative to leaf samples. The complete RT-qPCR dataset, including mean Ct values, technical standard deviations (SD), ΔCt, ΔΔCt, and relative expression values, is provided in Supplementary Table S1. The SD reported for RT-qPCR represents technical variation among the three Ct measurements and not variation among biological replicates. Oil content, PE concentration, and HHV data are also presented as means ± SD of three independently collected biological seed samples per genotype (*n* = 3). HHV was evaluated descriptively using the mean and SD; no inferential statistical comparison among genotypes was performed. For HHV, the standard error of the mean (SEM) was calculated as SD/√*n* and is reported in Supplementary Table S3, whereas the error bars in Fig. 7 represent SD. FA composition was analyzed separately at each fruit developmental stage using stage-specific PCA and HCA, including silhouette-based selection of the number of HCA clusters, as described in the preceding subsection. Univariate statistical analyses were performed using Sisvar (Ferreira 2014), whereas multivariate analyses were conducted in R (R Core Team 2026).

## Results

### Gene expression analysis

All primer pairs produced single amplicons of the expected sizes: *KASIII* (174 bp), *MFP2* (61 bp), *JcBAHD-AT* (112 bp), and *ubiquitin* (150 bp), as detailed in Table 3. Amplification specificity was further confirmed by melting-curve analysis, which revealed single, well-defined peaks for all targets, indicating the absence of nonspecific products or primer–dimer formation. The efficiency analysis demonstrated that all primer pairs exhibited amplification efficiencies within the acceptable range (83–100%), with high linearity (R^2^ ≥ 0.98), supporting their suitability for relative gene expression analysis using RT-qPCR.

**Fig. 2:**
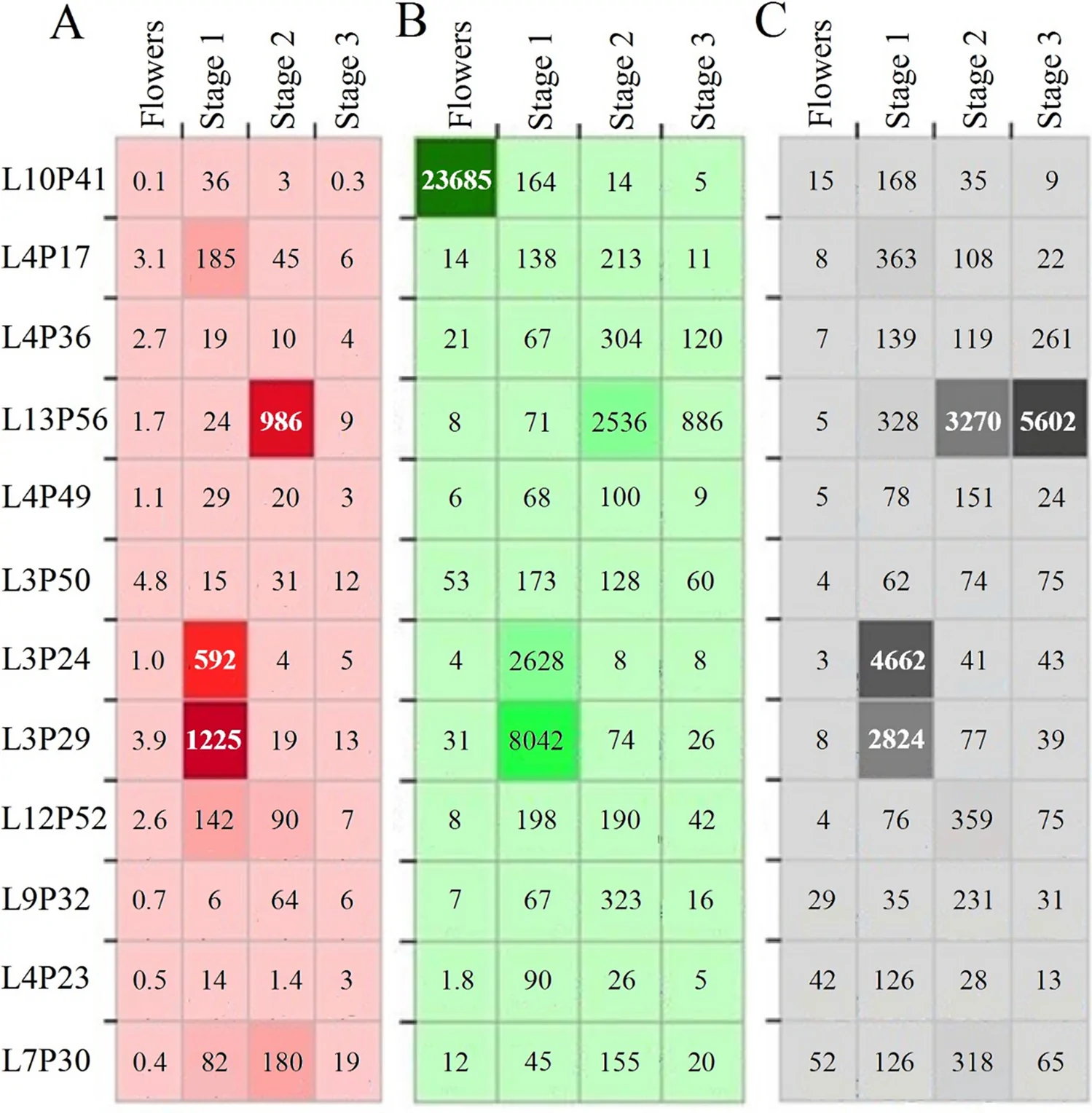
Heatmap visualization of the relative expression of *MFP2* (red, **A**), *KASIII* (green, **B**), and *JcBAHD-AT* (grayscale, **C**) in flowers and developing seeds of 12 *Jatropha curcas* genotypes. Relative expression was calculated using the 2⁻ΔΔCt method, with leaf samples used as the calibrator. RT-qPCR reactions were performed in technical triplicate for each genotype–tissue combination, and the mean Ct value of the technical replicates was used to calculate relative expression. Numerical values within cells represent approximate fold changes relative to the leaf calibrator. Darker shading indicates higher relative expression within each panel. Mean Ct values, technical-replicate standard deviations (SDs), ΔCt, ΔΔCt, and relative expression values are provided in Supplementary Table S1. The reported SD represents variation among technical replicates and not biological variability

The relative expression profiles of the three target genes across genotypes and tissues are presented in Fig. 2. Relative transcript abundance varied widely among genotypes, tissues, and developmental stages, with the highest values generally observed in developing seeds, although pronounced expression in flowers was observed for *KASIII* in L10P41. Detailed mean Ct values, technical-replicate SDs, ΔCt, ΔΔCt, and relative expression values calculated using the 2⁻ΔΔCt method are provided in Supplementary Table S1.

*MFP2* exhibited its highest expression in developing seeds, particularly at Stage 1 (S1) in genotypes L3P29 and L3P24, with transcript levels approximately 1225- and 592-fold relative to leaves, respectively. In genotype L13P56, *MFP2* reached its highest relative expression at Stage 2 (S2), with an increase of approximately 986-fold relative to the leaf calibrator (Fig. 2A). In contrast, *KASIII* showed its highest relative transcript abundance in the flowers of genotype L10P41, reaching approximately 23,685-fold relative to the leaf calibrator. Among developing-seed samples, *KASIII* showed markedly higher relative expression at S1 in genotypes L3P29 and L3P24 (approximately 8042- and 2628-fold, respectively) and at S2 in genotype L13P56 (approximately 2536-fold), whereas expression in the remaining genotypes remained comparatively low (Fig. 2B). *JcBAHD-AT* displayed a distinct temporal expression pattern. In genotype L13P56, relative transcript abundance increased progressively during seed development, rising from approximately 328-fold at S1 to 3270-fold at S2 and 5602-fold at S3 (Fig. 2C). In contrast, genotypes L3P24 and L3P29 exhibited high relative expression at S1 (approximately 4662- and 2824-fold, respectively), followed by a marked decline at S2 to approximately 40- and 80-fold, respectively, and S3 to approximately 40-fold in both genotypes. Overall, the three target genes exhibited distinct genotype- and developmental stage-dependent expression patterns, demonstrating substantial variation in transcript abundance during seed development**.**

### Oil and phorbol ester concentration

**Fig. 3:**
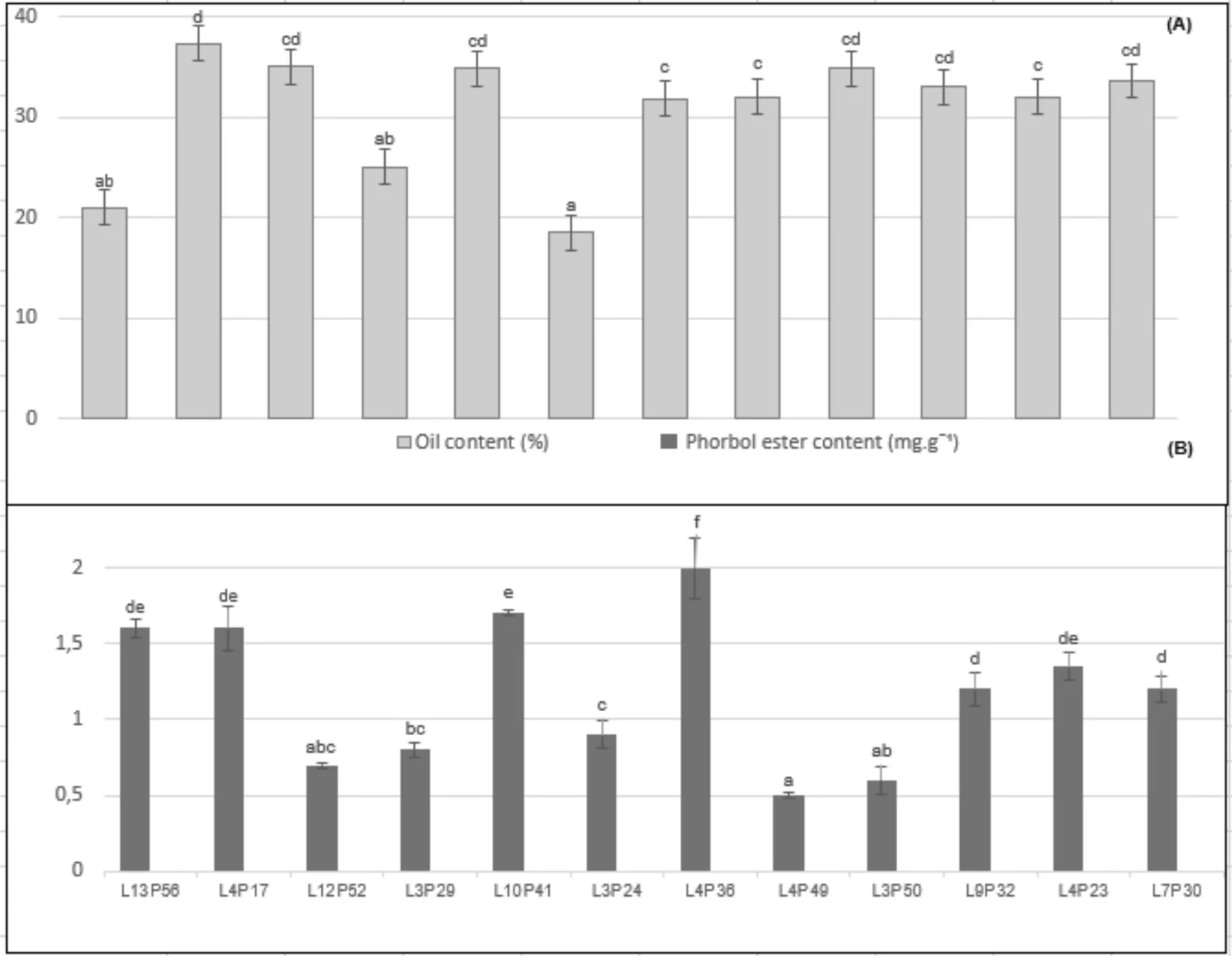
Oil content (%, **A**) and PE concentration (mg g⁻^1^, **B**) in mature seeds (41–45 DAP) of contrasting elite ***J. curcas*** genotypes. Bars represent means ± standard deviation (SD) of three independently collected biological seed samples (*n* = 3). Different letters indicate significant differences among genotypes according to the Scott–Knott test (*P* ≤ 0.05)

Figure 3 shows the oil content and PE concentration of mature seeds (41–45 DAP) from elite *J. curcas* genotypes previously selected by the IAC breeding program for contrasting agronomic and/or chemical traits (Table 1). Considerable genotypic variation was observed for both traits. Oil content rangedfrom 18.5% to 37.2%, whereas PE concentration ranged from 0.5 to 2.0 mg g⁻^1^. Genotypes with contrasting combinations of oil content and PE concentration were identified. L12P52 and L4P49 combined relatively high oil content with comparatively low PE concentrations, whereas L4P17 exhibited the highest oil content (37.2%) and L4P36 showed the highest PE concentration (2.0 mg g⁻^1^).

### FA composition

**Fig. 4:**
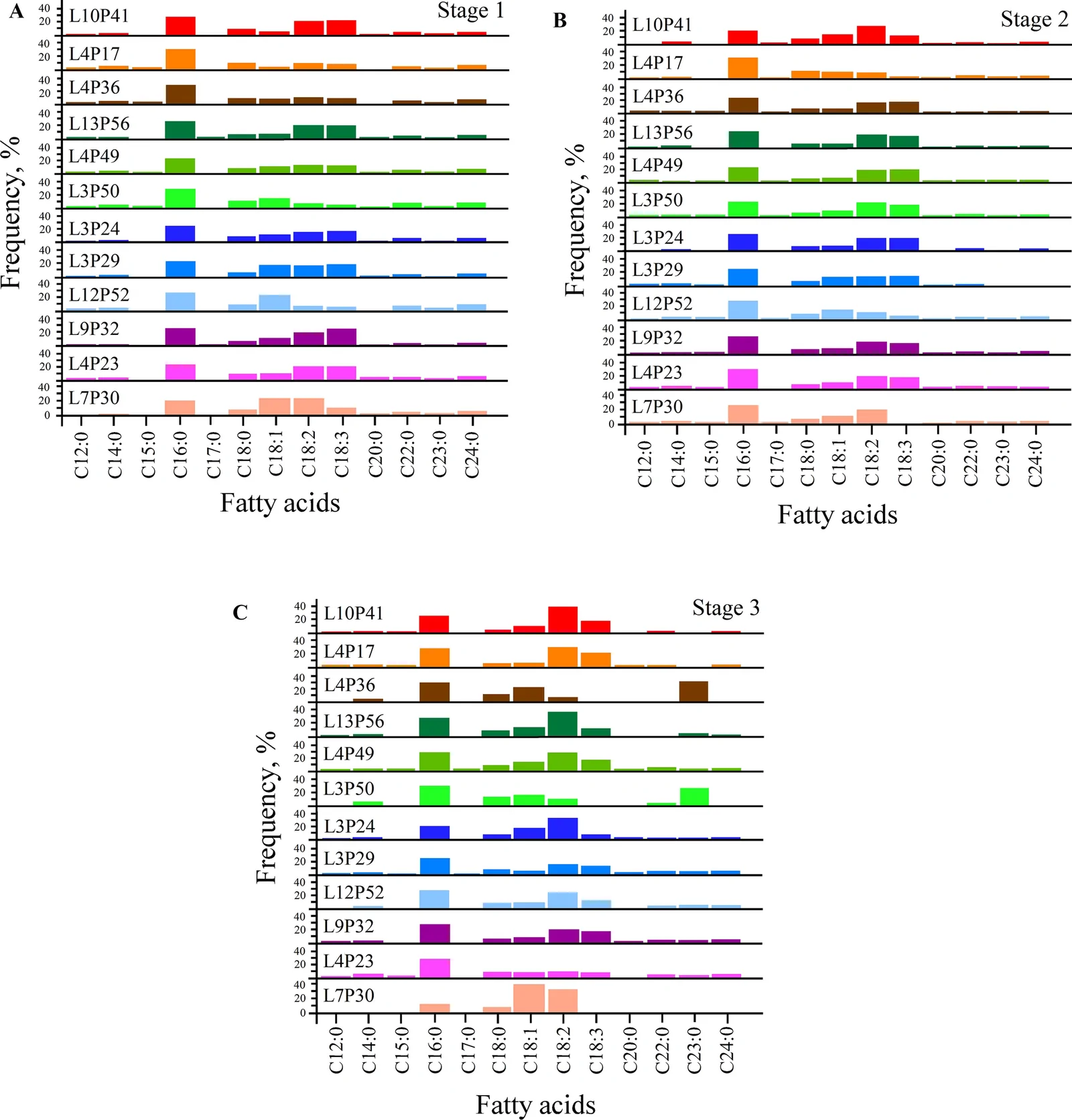
Fatty acid (FA) composition of *J. curcas* seeds at three developmental stages. **A** Stage 1 (fruit length 10–15 mm; ~15 DAP), **B** Stage 2 (fruit length 20–25 mm; ~19 DAP), and **C** Stage 3 (fruit length 30–40 mm; ~23 DAP). Each horizontal panel represents one genotype, and colored bars indicate the relative abundance (% chromatographic peak area) of individual FAs determined by gas chromatography with flame ionization detection (GC-FID). Complete FA composition data (means ± SD of three independently collected biological samples, *n* = 3) are provided in Supplementary Table S2

The FA composition of *J. curcas* seeds throughout fruit development was determined by gas chromatography with flame ionization detection (GC-FID). Marked variation in the relative abundance of individual FAs was observed among genotypes and developmental stages, with palmitic (C16:0), oleic (C18:1ω9), linoleic (C18:2ω6), and α-linolenic (C18:3ω3) acids representing the major components of the FA profiles. The relative distribution (% area) of individual FAs is presented in Fig. 4A–C, whereas the complete FA composition dataset for each genotype is provided in Supplementary Table S2. At Stage 1 (S1), palmitic acid (C16:0) was the predominant saturated FA in all genotypes, whereas oleic (C18:1ω9), linoleic (C18:2ω6), and α-linolenic (C18:3ω3) acids represented the major unsaturated FAs. Genotypes L3P29 and L12P52 exhibited comparatively higher proportions of oleic acid than the remaining genotypes (Fig. 4A). At Stage 2 (S2), palmitic acid remained predominant, although its relative abundance declined in some genotypes, particularly L10P41. Oleic acid showed genotype-dependent changes, increasing markedly in L10P41 while decreasing slightly in most of the remaining genotypes. Linoleic acid generally increased, whereas α-linolenic acid displayed contrasting genotype-specific trends. Minor amounts of cis-11-eicosenoic acid (C20:1ω11) and heneicosanoic acid (C21:0) were detected only in a limited number of genotypes (Fig. 4B). During Stage 3 (S3), genotype-specific differences became more pronounced. Capric (C10:0) and undecanoic (C11:0) acids were detected exclusively in genotype L3P24, which also exhibited the lowest oil content (Fig. 3). Compared with earlier stages, the relative abundance of oleic acid increased in genotypes L7P30, L10P41, and L4P36, whereas linoleic acid increased in several genotypes. In contrast, α-linolenic acid generally declined during seed maturation, indicating progressive remodeling of FA composition (Fig. 4C). Overall, these results showed substantial genotype-dependent variation in FA composition throughout seed development, characterized by dynamic changes in the relative abundance of saturated, monounsaturated, and polyunsaturated FAs during fruit maturation.

### Principal component, hierarchical cluster and heatmap analyses of FA composition

**Fig. 5:**
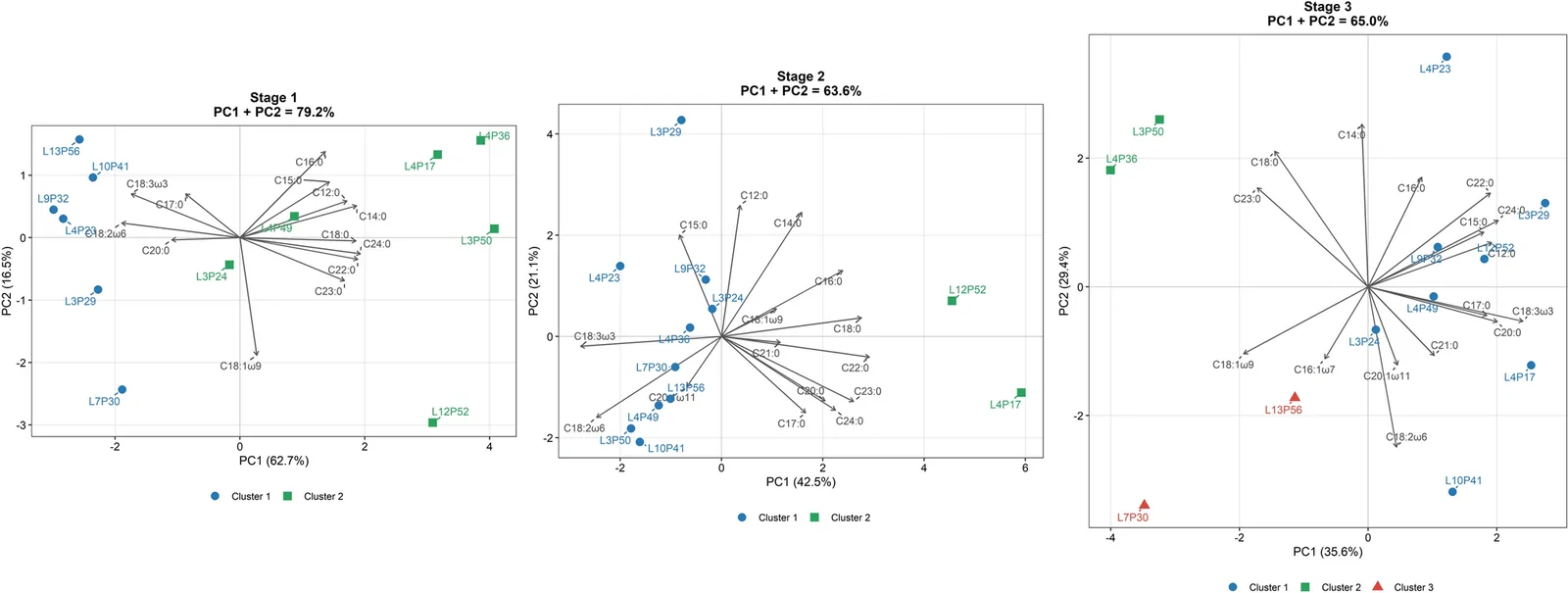
Principal component analysis (PCA) of fatty acid (FA) composition in twelve *J. curcas* genotypes during seed development. **A** Stage 1 (fruit length 10–15 mm; ~15 DAP), **B** Stage 2 (fruit length 20–25 mm; ~19 DAP), and **C** Stage 3 (fruit length 30–40 mm; ~23 DAP). PCA was performed separately for each developmental stage using the relative abundance (%) of identified FAs after mean-centering and unit variance scaling. Biplots display genotype scores and FA loading vectors. Arrows indicate the direction and relative contribution of each FA to the first two principal components. Colored symbols represent stage-specific genotype groups identified by hierarchical clustering analysis (HCA), with two clusters at Stages 1 and 2 and three clusters at Stage 3. Percentages on the axes indicate the proportion of total variance explained by the first two principal components (PC1 and PC2). The number of FA variables retained after stage-specific preprocessing was 13, 15, and 16 for Stages 1, 2, and 3, respectively. C12:0, lauric acid; C14:0, myristic acid; C15:0, pentadecanoic acid; C16:0, palmitic acid; C16:1ω7, palmitoleic acid; C17:0, margaric acid; C18:0, stearic acid; C18:1ω9, oleic acid; C18:2ω6, linoleic acid; C18:3ω3, α-linolenic acid; C20:0, arachidic acid; C20:1ω11, gondoic acid; C21:0, heneicosanoic acid; C22:0, behenic acid; C23:0, tricosanoic acid; C24:0, lignoceric acid

**Fig. 6:**
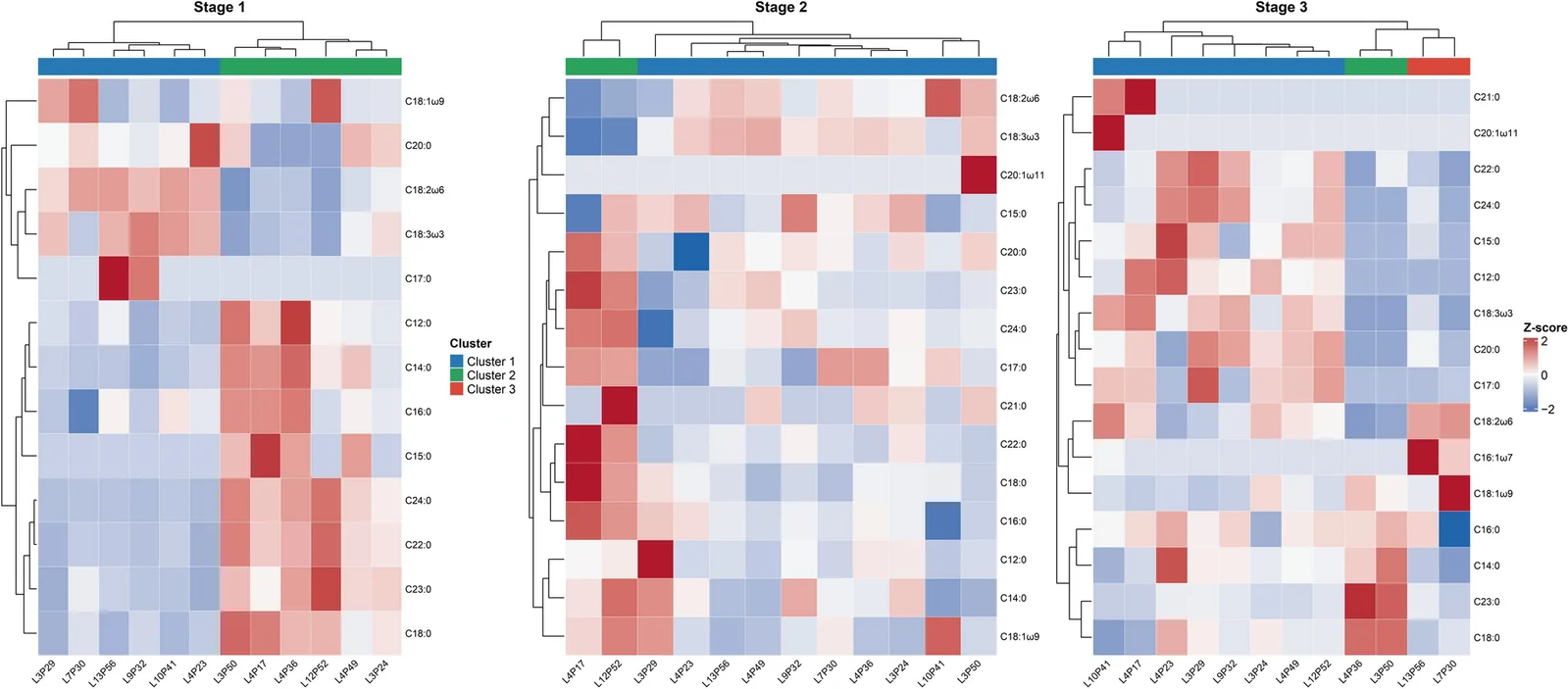
Hierarchical clustering analysis (HCA) and heatmap of fatty acid (FA) composition in twelve *J. curcas* genotypes during seed development. **A** Stage 1 (fruit length 10–15 mm; ~15 DAP), **B** Stage 2 (fruit length 20–25 mm; ~19 DAP), and **C** Stage 3 (fruit length 30–40 mm; ~23 DAP). HCA was performed separately for each developmental stage using the same standardized FA dataset employed for PCA. Clustering was based on Euclidean distance and Ward's minimum variance method (Ward.D2). The number of clusters was selected based on the highest average silhouette width among solutions ranging from *k* = 2 to *k* = 5, resulting in two clusters at Stages 1 and 2 and three clusters at Stage 3. Heatmaps display row-wise standardized FA abundances (Z-scores), where red indicates relatively higher abundance and blue indicates relatively lower abundance. Dendrograms illustrate genotype similarity based on FA composition, and cluster colors correspond to the HCA-defined groups represented in the PCA plots (Fig. 5)

PCA revealed distinct patterns of variation in FA composition among *J. curcas* genotypes at each developmental stage (Fig. 5A–C). HCA, performed using the same standardized FA dataset, resolved two genotype clusters at Stages 1 and 2 and three clusters at Stage 3 (Fig. 6A–C). The heatmaps further illustrated stage-specific differences in the relative abundance of individual FAs and the FA profiles associated with the HCA-defined genotype groups.

At Stage 1, PC1 and PC2 explained 62.7% and 16.5% of the total variance, respectively, accounting jointly for 79.2% (Fig. 5A). PC1 was positively associated mainly with C24:0, C22:0, C14:0, and C18:0 and negatively associated with C18:2ω6 and C18:3ω3. Oleic acid (C18:1ω9) showed its strongest contribution along PC2, with a negative loading. HCA resolved two equally sized clusters: Cluster 1 comprised L10P41, L13P56, L3P29, L9P32, L4P23, and L7P30, whereas Cluster 2 comprised L4P17, L4P36, L4P49, L3P50, L3P24, and L12P52 (Fig. 6A). At Stage 2, PC1 and PC2 explained 42.5% and 21.1% of the variance, respectively, accounting jointly for 63.6% (Fig. 5B). Positive PC1 loadings were strongest for C22:0, C18:0, C23:0, and C16:0, whereas C18:3ω3 and C18:2ω6 showed strong negative loadings. Variation along PC2 was associated particularly with C12:0, C14:0, and C15:0. HCA separated L4P17 and L12P52 into Cluster 2, whereas the remaining ten genotypes formed Cluster 1 (Fig. 6B). At Stage 3, PC1 and PC2 explained 35.6% and 29.4% of the total variance, respectively, accounting jointly for 65.0% (Fig. 5C). C18:3ω3, C24:0, C20:0, C12:0, and C22:0 showed strong positive associations with PC1, whereas C18:1ω9, C23:0, and C18:0 were associated with its negative direction. Along PC2, C14:0 showed the strongest positive loading, followed by C18:0 and C16:0, whereas C18:2ω6 showed the strongest negative loading. HCA resolved three groups: Cluster 1 comprised L10P41, L4P17, L4P49, L3P24, L3P29, L12P52, L9P32, and L4P23; Cluster 2 comprised L4P36 and L3P50; and Cluster 3 comprised L13P56 and L7P30 (Fig. 6C). Overall, the multivariate analyses revealed developmental changes in both the sources of FA variation and the grouping of genotypes. The first two PCs accounted for the highest proportion of variance at Stage 1 (79.2%), followed by Stage 3 (65.0%) and Stage 2 (63.6%), while HCA supported two genotype groups at Stages 1 and 2 and three groups at Stage 3.

### Higher heating value

**Fig. 7:**
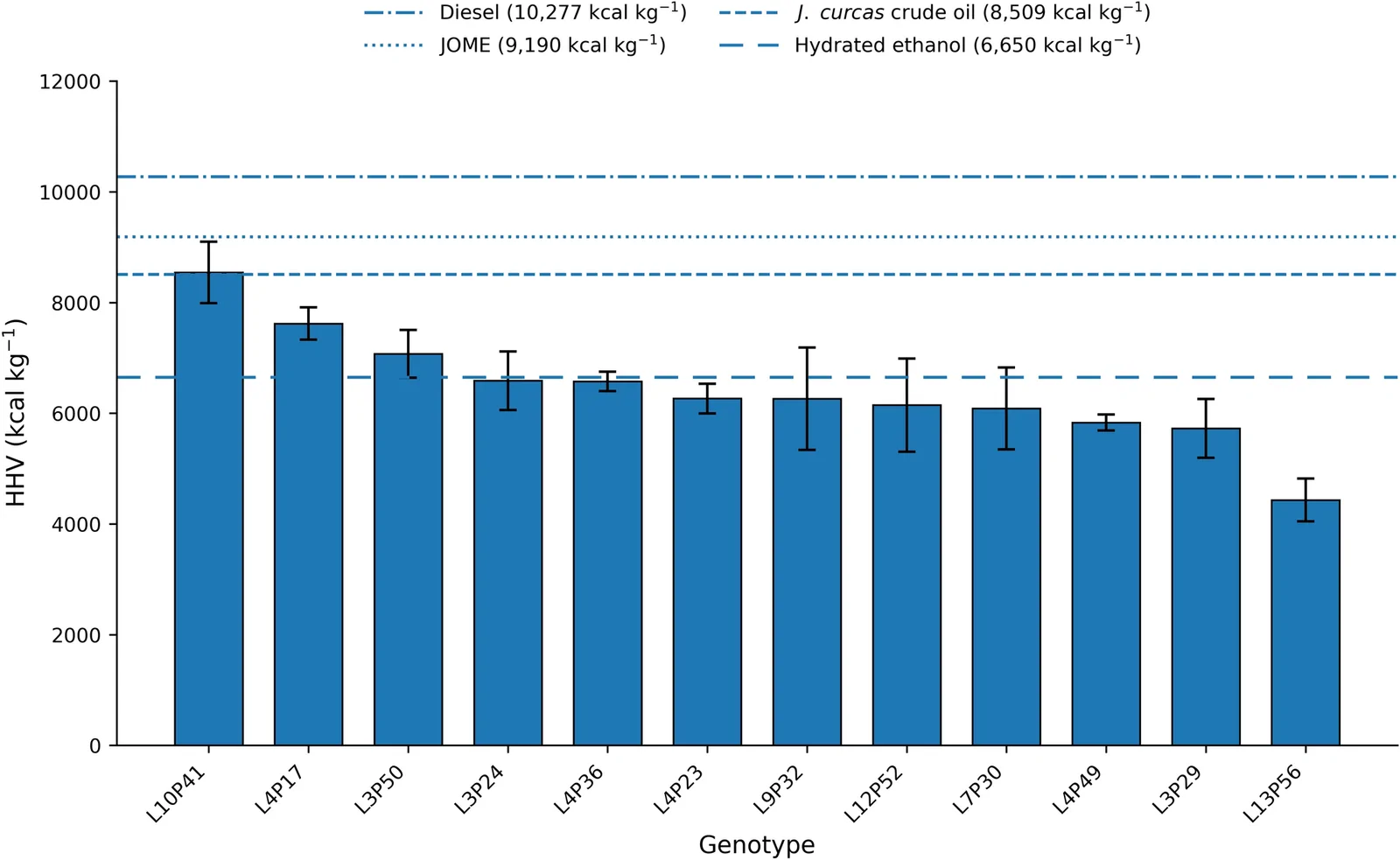
Higher heating value (HHV; kcal kg⁻^1^) of crude oil extracted from mature seeds of contrasting *Jatropha curcas* genotypes. Bars represent mean values ± standard deviation (SD) of three biological replicates (*n* = 3). Genotypes are arranged in decreasing order of mean HHV. Horizontal lines indicate reference HHV values for hydrated ethanol (6650 kcal kg⁻^1^), *J. curcas* crude oil (8509 kcal kg⁻^1^), *J. curcas* oil methyl ester (JOME; 9190 kcal kg⁻^1^), and diesel (10,277 kcal kg⁻^1^). The value for hydrated ethanol was obtained from the *Energy Balance of the State of São Paulo 2024* (São Paulo 2024), whereas those for *J. curcas* crude oil, JOME, and diesel were reported by Banapurmath et al. (2012)

HHV varied considerably among genotypes, with a mean value of 6429 kcal kg⁻^1^ (Supplementary Table S3). Genotypes L10P41, L4P17, and L3P50 exhibited HHV values exceeding 7000 kcal kg⁻^1^, with L10P41 showing the highest value (8544.12 kcal kg⁻^1^; Fig. 7). In contrast, L13P56 presented the lowest HHV (4433.71 kcal kg⁻^1^), followed by L3P29 (5727.83 kcal kg⁻^1^). The remaining genotypes showed intermediate values, ranging from 5832.50 kcal kg⁻^1^ in L4P49 to 6588.55 kcal kg⁻^1^ in L3P24 (Fig. 7). L10P41 exhibited an HHV slightly higher than the reference value reported for *J. curcas* crude oil (8509 kcal kg⁻^1^), whereas L4P17 and L3P50 exceeded that of hydrated ethanol (6650 kcal kg⁻^1^). None of the evaluated genotypes reached the reference HHV values reported for *J. curcas* oil methyl ester (JOME; 9190 kcal kg⁻^1^) or diesel (10,277 kcal kg⁻^1^) (Fig. 7).

## Discussion

The establishment of *J. curcas* as a sustainable bioenergy crop depends on breeding strategies aimed at increasing oil content, reducing PE content, and optimizing FA composition and HHV. The IAC breeding program has developed genetically diverse elite genotypes through interspecific hybridization and selection, as demonstrated by ISSR marker analysis (Díaz et al. 2017) and extensive phenotypic variation in oil, FA, and PE contents (Ferrari et al. 2009; de Argollo Marques et al. 2013). This diversity provided a suitable framework for investigating relationships among metabolic traits, gene expression, and bioenergy-related phenotypes during seed development.

Oil accumulation in *J. curcas* involves multiple genetic and metabolic processes associated with FA biosynthesis and lipid metabolism (Costa et al. 2010; Li-Beisson et al. 2013), whereas PE biosynthesis has been reported as a maternally controlled dominant monogenic trait (King et al. 2013). Within this framework, the expression patterns of *KASIII*, *MFP2*, and *JcBAHD-AT* may provide complementary information on FA biosynthesis, lipid turnover, and specialized metabolism during seed development. Because these processes involve interconnected metabolic networks, the gene–metabolite relationships identified here should be interpreted as associative rather than causal and require further functional validation.

### Developmental dynamics of fatty acid composition and oil quality

FA composition changed markedly throughout seed development, consistent with developmental remodeling of lipid composition previously reported in developing *J. curcas* seeds (Annarao et al. 2008). Although palmitic acid (C16:0) remained the predominant saturated FA, the relative abundances of oleic (C18:1), linoleic (C18:2), and α-linolenic (C18:3) acids varied among genotypes and developmental stages. These shifts highlight substantial genotype-dependent variation in FA composition and are consistent with previous reports of broad variation in FA composition and oil quality among *J. curcas* accessions (Alburquerque et al. 2017; Kumar and Das 2018) and during seed filling (Gu et al. 2012).

Multivariate analyses further demonstrated that genotype differentiation based on FA composition was developmentally dynamic, with both the variables contributing most strongly to PCA separation and the HCA-defined genotype groups changing across stages. This stage-dependent restructuring of FA profiles reinforces the importance of considering seed developmental stage when characterizing lipid-related diversity and selecting elite *J. curcas* germplasm for oil-quality traits.

A descriptive tendency toward lower HHV was observed in genotypes with higher relative abundances of saturated and very-long-chain FAs compared with genotypes characterized by higher relative abundance of oleic acid. More broadly, FA chain length and degree of unsaturation are known to influence the fuel properties and energy content of fatty-acid-derived fuels (Knothe 2008; Ramos et al. 2009). Notably, L3P24 exhibited relatively high HHV despite its comparatively low oil content, suggesting that energy performance cannot be inferred from total oil content alone and may also be associated with differences in oil composition. L10P41 emerged as a promising bioenergy ideotype**,** combining elevated oleic acid content, high oil accumulation, and the highest HHV measured in this study**.** These findings support the combined consideration of FA composition and oil content when selecting *J. curcas* germplasm for bioenergy applications. Although HHV remained below that of fossil diesel, the highest values observed among the elite *J. curcas* genotypes exceeded that of hydrated ethanol and approached values previously reported for *J. curcas* seed oil (Fig. 7), reinforcing the potential of selected genotypes as renewable bioenergy feedstocks (São Paulo 2024; Banapurmath et al. 2012).

### Gene expression and lipid metabolic regulation

The integration of gene expression with FA profiles revealed genotype- and stage-dependent associations between lipid composition and the expression of *KASIII* and *MFP2*, consistent with their respective roles in FA biosynthesis and turnover. Although these relationships are associative rather than causal, they provide a molecular framework for interpreting differences in lipid accumulation and oil quality among elite *J. curcas* genotypes.

Higher *MFP2* transcript abundance, particularly at S1 and S3, tended to occur in  genotypes characterized by greater proportions of saturated and very-long-chain FAs. *MFP2* encodes a multifunctional peroxisomal enzyme involved in the hydration and dehydrogenation steps of FA β-oxidation (Arent et al. 2010). Because β-oxidation participates in FA turnover and can influence the availability of acyl substrates for triacylglycerol (TAG) accumulation (Eastmond and Graham 2000; Rylott et al. 2006; Li-Beisson et al. 2013), the association between elevated *MFP2* expression and lower oil accumulation observed in some genotypes may reflect differences in the balance between lipid turnover and storage. Particularly relevant for breeding, the association observed at S1 suggests that *MFP2* could be investigated as a candidate molecular indicator for early screening of genotypes with contrasting oil-accumulation potential, thereby assisting the early identification of less favorable genotypes before mature-seed phenotyping. The recurrence of this association at S3 further supports its potential biological relevance. However, its predictive value and utility for selection will require validation in larger breeding populations and functional studies.

*KASIII* catalyzes the initial condensation step of plastidial de novo FA biosynthesis, providing acyl intermediates for subsequent chain elongation (Li-Beisson et al. 2013). Previous characterization of *JcKASIII* showed broad tissue expression in *J. curcas*, with increasing expression during seed development (Li et al. 2008). The pronounced floral *KASIII* expression observed in L10P41 may indicate genotype-specific regulation of de novo FA biosynthesis during reproductive development. This interpretation is biologically plausible given the substantial lipid metabolic activity required during reproductive processes, including pollen germination and pollen tube growth (Hernández et al. 2020). However, the co-occurrence of high floral *KASIII* expression, high fruit yield, and high oil content in L10P41 does not establish a functional relationship among these traits. Functional characterization has shown that *JcKASIII* overexpression in *Arabidopsis* alters seed FA composition, including increased palmitic acid content and the C16:C18 ratio (Yu et al. 2015). The absence of a uniform relationship between *KASIII* expression and FA composition across the genotypes evaluated here indicates that final FA profiles likely result from coordinated regulation of de novo FA synthesis together with downstream elongation and desaturation processes rather than from *KASIII* expression alone.

### Integration of lipid and specialized diterpenoid metabolism

*JcBAHD-AT* represents a candidate gene potentially associated with specialized diterpenoid metabolism in *J. curcas*. The gene (Jcr4S00504.110) shares approximately 90% sequence similarity with a predicted 3′-*N*-debenzoyl-2′-deoxytaxol N-benzoyltransferase from *Ricinus communis*. BAHD acyltransferases comprise a functionally diverse enzyme family involved in specialized metabolism, and members of this family participate in late acylation steps of taxane biosynthesis in *Taxus* species (D'Auria 2006; Long et al. 2008). BAHD acyltransferases have also been identified as candidate enzymes associated with diterpenoid modification in Euphorbiaceae (King et al. 2014). However, no evidence supports taxane biosynthesis in *J. curcas*; therefore, the sequence-based annotation of *JcBAHD-AT* remains putative and does not imply conservation of biochemical function. Rather, its genotype- and developmental stage-dependent expression supports a potential role in lineage-specific diterpenoid metabolism.

Within Euphorbiaceae, specialized diterpenoid metabolism includes casbene-derived pathways associated with PE biosynthesis in *J. curcas* (Nakano et al. 2012; King et al. 2014). In the present study, *JcBAHD-AT* expression varied markedly among genotypes and developmental stages and coincided with variation in PE accumulation and lipid-related traits. Although *KASIII* does not directly participate in diterpenoid biosynthesis, the genotype- and stage-dependent expression of genes associated with FA biosynthesis and turnover (*KASIII* and *MFP2*), together with variation in *JcBAHD-AT* expression, is consistent with broader differences in carbon allocation between primary and specialized metabolism. This interpretation is biologically plausible because acetyl-CoA represents an important metabolic node connecting FA biosynthesis with the mevalonate pathway, while isoprenoid precursors derived from both the mevalonate and plastidial MEP pathways contribute to multiple branches of specialized metabolism, including carotenoid and diterpenoid biosynthesis (King et al. 2014; Song et al. 2022). Experimental evidence for potential trade-offs in precursor allocation comes from *Aurantiochytrium* sp., in which overexpression of the *KASIII*-like gene *YxwZ3* increased carotenoid production while reducing FA accumulation and the expression of FA biosynthetic genes; the authors proposed preferential allocation of malonyl-CoA toward the mevalonate pathway as a possible explanation (Song et al. 2022). Similarly, integrated transcriptomic and metabolomic analyses in the woody oil species *Idesia polycarpa* revealed coordinated variation among oil content, FA composition, and specialized metabolites and suggested substrate-allocation trade-offs between FA and pigment biosynthetic pathways (Song et al. 2026). At a broader regulatory level, *SnRK1*-mediated signaling integrates cellular carbon and energy status with metabolic regulation, providing a mechanistic framework through which resource availability may influence allocation among competing metabolic processes (Han et al. 2024). Sugar availability can also interact with hormonal and developmental signaling, linking carbon status to resource allocation and plant development (Liu et al. 2024). Together, these studies support the biological plausibility of coordinated regulation between lipid and specialized metabolism; however, the relationships observed in *J. curcas* remain inferential because metabolic fluxes and the relevant precursor pools were not directly measured.

The biological function of *JcBAHD-AT* remains to be experimentally validated because this study did not include enzymatic assays or targeted diterpenoid profiling. Future integration of functional characterization, targeted metabolomics, and metabolic-flux analyses will be required to determine whether variation in *JcBAHD-AT* expression is accompanied by coordinated changes in lipid and specialized diterpenoid metabolism. Such integrative approaches combining molecular and metabolic information can help resolve complex gene–metabolite–phenotype relationships (Raza et al. 2025).

### Integrated molecular and metabolic features of the bioenergy ideotype

Overall, the present results support a model in which superior bioenergy performance in *J. curcas* reflects the coordinated contribution of multiple metabolic processes rather than the action of individual genes. Considered together, the genotype- and stage-dependent expression patterns of *KASIII*, *MFP2*, and *JcBAHD-AT*, the developmental variation in FA composition characterized by stage-specific PCA and its complementary HCA, and variation in oil content, PE accumulation, and HHV provide complementary molecular, metabolic, and energetic information for distinguishing bioenergy phenotypes. Importantly, these relationships should be interpreted as an integrated phenotypic framework rather than evidence of direct regulatory links among these traits.

Among the evaluated genotypes, L10P41 emerged as a favorable bioenergy ideotype, combining elevated oleic acid content, high oil accumulation, and the highest HHV measured in this study (8544.12 kcal kg⁻1), together with distinct expression patterns of the candidate genes and favorable agronomic attributes, including high fruit yield (Table 1). Notably, its HHV was slightly higher than the reference value reported for crude *J. curcas* oil (8509 kcal kg⁻1), although it remained below those reported for *J. curcas* oil methyl ester (JOME; 9190 kcal kg⁻1) and diesel (10,277 kcal kg⁻1) (Banapurmath et al. 2012; Fig. 7). Together, these traits reinforce L10P41 as a promising bioenergy ideotype and illustrate the value of integrating multiple selection criteria rather than relying on a single trait.

Previous studies have demonstrated substantial variation among *J. curcas* accessions in oil content, FA composition, and oil-quality traits, highlighting the value of this diversity for germplasm selection and prebreeding (Alburquerque et al. 2017; Kumar and Das 2018). The present findings extend this framework by showing that the multivariate structure of FA composition was developmentally dynamic, with genotype relationships changing across seed developmental stages, while molecular, metabolic, energetic, and agronomic traits provide complementary criteria for identifying elite germplasm. Together, these stage-dependent patterns suggest that developmental stage should be considered when defining phenotypic and molecular assessment strategies. However, the gene–trait relationships identified here remain associative and require functional validation before candidate genes can be considered for breeding applications. Future integration of functional genomics, targeted metabolomics, and metabolic flux analyses should further clarify the regulatory networks underlying carbon partitioning, lipid composition, and specialized metabolism in *J. curcas*.

## Conclusion

This study revealed substantial genotype- and developmental stage-dependent variation in FA composition and candidate-gene expression in *J. curcas*. Stage-specific PCA, complemented by HCA, further demonstrated that the multivariate structure of FA composition and genotype relationships changed across seed development. Among the evaluated genotypes, L10P41 emerged as a promising bioenergy ideotype, combining high oleic acid content, relatively high oil content, and high HHV, whereas L4P49 and L12P52 represent valuable genetic resources for breeding strategies targeting high oil content and lower PE levels. Overall, integrating molecular, metabolic, and energetic traits provides a useful framework for identifying elite *J. curcas* germplasm, although functional validation of the candidate genes and further integration with targeted metabolomics and metabolic flux analyses will be required to clarify the regulatory mechanisms underlying these associations and support their future application in bioenergy-oriented breeding.

## Supplementary Information

Below is the link to the electronic supplementary material.

Supplementary file1 (XLSX 24 KB)

Supplementary file2 (XLSX 17 KB)

Supplementary file3 (XLSX 10 KB)

## Data Availability

The datasets generated and/or analyzed during the current study are available from the corresponding author on reasonable request.

## Abbreviations

ACP: Acyl carrier protein
BAHD: Acyltransferase family named after BEAT, AHCT, HCBT, and DAT
Ct: Cycle threshold
DAP: Days after pollination
FA: Fatty acid
HCA: Hierarchical cluster ﻿analysis
PCA: Principal component analysis
HHV: Higher heating value
*JcBAHD-AT*: Putative *Jatropha curcas* BAHD acyltransferase
*KASIII*: 3-Ketoacyl-ACP synthase III
*MFP2*: Multifunctional protein 2
PE: Phorbol ester
